# Relative telencephalon size does not affect collective motion in the guppy (*Poecilia reticulata*)

**DOI:** 10.1093/beheco/arae033

**Published:** 2024-05-03

**Authors:** Annika Boussard, Mikaela Ahlkvist, Alberto Corral-López, Stephanie Fong, John Fitzpatrick, Niclas Kolm

**Affiliations:** Department of Zoology, Stockholm University, Svante Arrhenius väg 18B, 106 91 Stockholm, Sweden; Department of Zoology, Stockholm University, Svante Arrhenius väg 18B, 106 91 Stockholm, Sweden; Department of Ecology and Genetics, Uppsala University, Norbyvägen 18D, 752 36 Uppsala, Sweden; Department of Zoology, Stockholm University, Svante Arrhenius väg 18B, 106 91 Stockholm, Sweden; Department of Zoology, Stockholm University, Svante Arrhenius väg 18B, 106 91 Stockholm, Sweden; Department of Zoology, Stockholm University, Svante Arrhenius väg 18B, 106 91 Stockholm, Sweden

**Keywords:** collective motion, guppy, telencephalon

## Abstract

Collective motion is common across all animal taxa, from swarming insects to schools of fish. The collective motion requires intricate behavioral integration among individuals, yet little is known about how evolutionary changes in brain morphology influence the ability for individuals to coordinate behavior in groups. In this study, we utilized guppies that were selectively bred for relative telencephalon size, an aspect of brain morphology that is normally associated with advanced cognitive functions, to examine its role in collective motion using an open-field assay. We analyzed high-resolution tracking data of same-sex shoals consisting of 8 individuals to assess different aspects of collective motion, such as alignment, attraction to nearby shoal members, and swimming speed. Our findings indicate that variation in collective motion in guppy shoals might not be strongly affected by variation in relative telencephalon size. Our study suggests that group dynamics in collectively moving animals are likely not driven by advanced cognitive functions but rather by fundamental cognitive processes stemming from relatively simple rules among neighboring individuals.

## Teaser

Collective motion occurs when animals coordinate their movement in schools, flocks, or herds. Using an experimental approach, we show that the size of the cognitive center in the vertebrate brain, the telencephalon, does not influence the ability to coordinate movement in single-sex guppy shoals artificially selected to have large or small telencephalon size. Advanced cognitive functions are likely not used during coordination of movements in guppy shoals.

## Introduction

Collective motion involves the coordinated movement of animal groups without centralized leadership, often seen in flocks of birds, schools of fish, and herds of mammals (Parrish et al. 2002; Couzin and Krause 2003; Sumpter 2010; Herbert-Read 2016). At the core of the study of collective motion is how individual-level behavior can lead to group-level patterns. Thus, collective motion emerges when the actions of each group member are influenced by the actions of their near neighbors (Couzin et al. 2002; Sumpter et al. 2012; Vicsek and Zafeiris 2012). With recent advances in analytical techniques, we now have a detailed understanding of the interaction rules that enable highly coordinated and synchronized movements of animal groups (Couzin et al. 2005; Herbert-Read et al. 2011; Katz et al. 2011; Pettit et al. 2013; Schaerf et al. 2021). These rules include, for instance, alignment and attraction with near neighbors to facilitate information transfer and repulsion to avoid overcrowding and collisions (Cousin et al. 2002; Vicsek and Zafeiris 2012; Herbert-Read 2016). Group members use these rules to transfer information about potential threats (Krause and Ruxton 2002) and feeding opportunities (Bazazi et al. 2012). Variations in how these interaction rules are used can affect collective motion (Sumpter et al. 2018; Kotrschal et al. 2020). And the use of interaction rules does indeed vary between both species and populations (Parrish et al. 1999; Huizinga et al. 2009; Herbert-Read 2016; Sumpter et al. 2018), as well as between individuals (Herbert-Read 2016; Jolles et al. 2017; Schaerf et al. 2021). This has been explained by a variety of intrinsic and extrinsic biological factors, such as predation pressure (Herbert-Read et al. 2017), age, and spatial position preferences (Herbert-Read 2016). For instance, more polarized groups are seen in older tadpoles of clawed frogs (*Xenopus laevis*), shrimps (*Paramesopodopsis rufa*), and squids (*Loligo opalescens*), than in their younger conspecifics (Herbert-Read 2016). And female guppies (*Poecilia reticulata*) from high-predation areas form larger and more cohesive shoals than females from low-predation areas (Herbert-Read et al. 2017). Variation in brain anatomy is another important factor that underlies substantial amounts of variation in social behavior across taxa (Barton 1996; Dunbar 1998; Reader and Laland 2002; Burish et al. 2004; Striedter 2005; Pollen et al. 2007; Stednitz et al. 2018; Triki et al. 2020). Yet, further investigations of intrinsic mechanisms underlying variation in collective motion are paramount to fully understand their evolution.

There are several reasons to assume that variations in brain anatomy affect collective motion. First, to determinate the locations of neighboring individuals, animals must integrate multiple sensory inputs such as visual, chemical, and tactical cues (Pitcher et al. 1976; Ballerini et al. 2008; Vicsek and Zafeiris, 2012; Strandburg-Peshkin et al. 2013; Rosenthal et al. 2015). Moreover, the location and behavior of several neighbors must be assessed simultaneously. For instance, starlings use the positions of up to 8 nearest neighbors to position themselves within the flock (Ballerini et al. 2008). Differential investment into specific brain regions associated with such information processing, for instance, telencephalon, optic tectum, and thalamus (Broglio et al. 2003, 2005; Salas et al. 2003; Butler and Hodos 2005), can thus result in more efficient tracking and response to neighbors’ movements, which in turn may facilitate coordinated behavior. Second, brain regions involved in aspects of higher cognitive abilities, such as the forebrain that controls decision-making and social cognition (Reader and Laland 2002; Schultz and Dunbar 2010; Triki et al. 2020), can potentially impact the ability to make rapid and adaptive decisions during collective motion. This could be crucial for avoiding collisions, responding to changes or threats in the environment or adjusting to the movement of the group (Pitcher et al. 1976; Couzin and Krause 2003; Couzin et al. 2005; Ballerini et al. 2008; Huizinga et al. 2009; Herbert-Read et al. 2011; Vicsek and Zafeiris 2012; Strandburg-Peshkin et al. 2013; Rosenthal et al. 2015). Third, brain regions associated with spatial cognition and navigation could play a role in maintaining a sense of direction and position within a moving group. Across vertebrates, the forebrain is involved in spatial cognition and navigation (Broglio et al. 2003, 2005; Butler and Hodos 2005). Thus, variation in forebrain structures may affect the ability to for instance compute distance to near neighbors or retain mental maps of the surroundings, which aids in navigating and aligning with group members during collective motion.

Collective motion in fish has been suggested to at least partly be processed by the ventral part of the telencephalon (Shinozuka and Watanabe 2004). Telencephalon-ablated goldfish showed reduced shoaling propensity and speed compared to controls (Shinozuka and Watanabe 2004). This suggests that the presence or absence of the telencephalon plays a crucial role in facilitating the ability of fish to coordinate their movements within a group. However, it is not clear how variation in the size of the telencephalon influences collective motion, particularly in an evolutionary context. Given the function of the telencephalon (Broglio et al. 2003, 2005; Salas et al. 2003; Shinozuka and Watanabe 2004; Butler and Hodos 2005), we would expect that fish with larger telencephalon size exhibit more coordinated and cohesive behavior, enhancing their ability to navigate and respond collectively to environmental stimuli. Whether variation in telencephalon size has any effect on collective motion has yet to be tested.

The Trinidadian guppy (*Poecilia reticulata*) is a commonly used evolutionary model system to test hypotheses on various aspects of brain evolution and collective motion (e.g. Herbert-Read et al. 2015, 2017; Kotrschal et al. 2018, 2020; Sumpter et al. 2018; Corral-López et al. 2023). In this study, we use guppies artificially selected for small and large relative telencephalon size to answer the question: *how do independent changes in relative telencephalon size affect collective motion?* After 5 generations of selection, a difference of approximately 10% in telencephalon volume between the telencephalon size selection lines has been established (Triki et al. 2023). By using high-resolution trajectory data on the movement of guppy shoals, we quantify how variation in relative telencephalon size affects several aspects of collective motion when exploring an open field in these selection lines. We predict to find a difference between large compared to small telencephalon size selected shoals. On the one hand, it could be that a larger relative telencephalon size enhances the ability to form aligned and cohesive shoals. On the other hand, it could be that an increase in telencephalon size might not affect collective motion positively, but a decrease in telencephalon size might instead have a negative effect. Under both scenarios, we would expect small telencephalon-size guppy shoals to form less aligned and cohesive shoals.

## Material and methods

### Model system

We conducted this study at the freshwater aquarium facilities at Stockholm University between March and April 2021. The fish used in this study were laboratory-breed descendants to wild-caught guppies from high-predation areas in the Quare River in Trinidad. The artificial selection started with 3 breeding stocks (hereafter replicates) that were up- vs down-selected for telencephalon size (i.e. 2 selection lines per replicate, resulting in 3 up-selected lines and 3 down-selected lines in total). The offspring of 75 breeding pairs per replicate with the 20% largest vs 20% smallest relative telencephalon volume (relative to the rest of the brain) were used as the next generation. After 5 generations of this selection procedure, an approximate 10% difference in telencephalon volume was observed in both sexes between the large and small telencephalon size selection lines (Fong et al. 2021; Triki et al. 2023). For details on the selection procedure, see Fong et al. (2021). Sexually mature fish were housed in stock tanks in groups of 8 to 9 females and 40 males, sorted by replicate and selection line. The laboratory was kept at a 12:12 h dark: light scheme and 24 ± 1 °C water temperature. All tanks were enriched with 2 cm light gravel, biological filter, snails (*Planorbis sp*), java moss (*Taxiphyllum sp.*), and/or artificial plants. Fish were fed 6 d per week with flake food and *Artemia nauplii* hatchlings interchangeably. Experiments were approved by the Stockholm animal research ethical board permit numbers (Dnr: N173/13, 223/15, N8/17 and 17362-2019).

### Behavioural assay

We assayed collective motion using a standard open-field test (OFT). Prior to the OFT, we collected 960 sexually mature males and females from stock tanks (we used visual detection of gonopodium length in males and gravid spot in females as sign of sexual maturity), equally distributed among sex, from 3 replicates of the small and large telencephalon size selection lines. We measured single-sex shoals of 8 individuals in the OFT, resulted in 120 shoals in total, i.e. 60 shoals from the large telencephalon size selection lines (30 female and 30 male shoals) and 60 shoals from the small telencephalon size selection lines (30 female and 30 male shoals). Hence, all the collected 960 fish were used in the experiment. Prior to assays, we housed shoals in 7 L holding tanks throughout the experiment. Visual contact between the tanks was allowed. We minimized observer bias by coding holding tanks with running numbers, labeled by a person unrelated to the experiments. During assays, we used four round white arenas with 55 cm in diameter, and with 3 cm water depth. We placed guppy shoals in the middle of the arena in an opaque white 15 cm PVC cylinder and left fish to acclimatize during 2 min. To minimize stress, fish were always transferred in bowls with water. We changed approximately half of the water in the arenas between trials to ensure that conspecific chemical cues remained relatively constant. Trials were run between 9.00 and 18.00 each day.

We recorded OFTs using Point Grey Grasshopper 3 cameras (FLIR Systems; resolution, 2048 pixels by 2048 pixels; frame rate, 25 Hz), placed above the arenas. The video recording started when the cylinders were lifted and continued for ten minutes with a 30-fps frame rate. The shoals were filmed in random order. We used idTracker (Pérez-Escudero et al. 2014) to track the movement of the shoals from recorded videos. The shoals were tracked from the 2nd to the 10th minute (sensu Kotrschal et al. 2018, 2020). We chose shoals of 8 guppies because it is the largest number of individuals the software can handle without comprising data efficiency and because it corresponds to shoal size in natural conditions (Croft et al. 2003a, 2003b). Data extraction was done in MATLAB 2020 following methods established in Kotrschal et al. (2018) and Kotrschal et al. (2020).

To quantify collective motion, we used the fine-grained tracking data to calculate group-level properties that characterize the structure and dynamics of guppy shoals (Herbert-Read et al. 2017; Kotrschal et al. 2018; Sumpter et al. 2018). These included alignment, nearest neighbor distance (henceforth attraction), and speed. Alignment is a key feature and an important prerequisite for collective motion to occur (Vicsek and Zafeiris 2012), since a group of animals orientated in different directions are not able to move coordinated and synchronized in the same direction. The attraction between near neighbors is an important force to keep groups cohesive (Herbert-Read et al. 2011; Vicsek and Zafeiris 2012). Alignment and attraction are mediated at least partly by changes in speed (Herbert-Read et al. 2011). Alignment was calculated as the median global alignment per guppy shoal, which measures the angular alignment (i.e. orientation of the head of the nearest neighbor) between all fish in the arena, across all frames, and is a dimensionless score. These calculations for the median global alignment in each frame were performed only when 6 out of the 8 shoal members had reliable tracks, following the optimization of our tracking protocol as described in Szorkovszky et al. (2018). If all fish are aligned in the same direction, the score is equal to one and decreases as fish are less aligned. To calculate attraction, the median distance in mm to the nearest neighbor across all frames was used. To measure speed [mm*s^−1^], we determined the median speed for all shoal members. This was achieved by calculating the first derivates of the x and y time series and then applying a smoothing process using a third-order Savitzky-Golay filer. In addition, we also measured meandering (i.e. absolute turning degree per second), spatial group spread (mm), and main sub-group size. These group-level properties influence the level of alignment in simulation and empirical studies and describe the spatial structure and dynamics in animal groups (Vicsek et al. 1995; Ballerini et al. 2008; Strömbom 2011). Spatial shoal spread and sub-group size influence collective motion, as less spread-out groups and larger groups are generally more aligned, while meandering describes group dynamics and is not expected to either increase or decrease with the level of alignment (Vicsek et al. 1995; Vicsek and Zafeiris 2012; Bagarti and Menon 2019). Meandering was calculated as the median across all fish per shoal for every frame. We calculated the spatial spread of each shoal as the mean global radius to the group centroid across all fish per shoal and all frames. The main sub-group size was determined as the mean number of fish of the largest sub-group with an interindividual distance of < 100 mm. Absolute body size was estimated from idTracker and calculated as the mean number of pixels per shoal. All measurements were averaged across all frames, individuals, and shoals per time period, i.e. from the 2nd to the 10th minute. Tracking data that did not present at least 16 consecutive tracked frames were disregarded in the calculations; these made up to 7.3% of the tracking data for all shoals. For full details on the calculations, see Sumpter et al. (2018) and Kotrschal et al. (2018).

Guppies artificially selected for relative brain size show differences in boldness in an open arena test (Kotrschal et al. 2014). Artificial selection on brain anatomy can thus affect the boldness/shyness axis in guppies. Previous results suggest that boldness is unaffected by artificial selection on relative telencephalon size (Fong 2020), but this has not been tested in a collective motion context and not in the specific generation used in this study. Variation in collective motion has previously been associated with variation in boldness in schooling fish (Jolles et al. 2017; Tang and Fu 2020). To investigate potential differences in boldness between the telencephalon size selection lines, we measured the distance to the arena center. In open-field tests, the distance to the arena center is sometimes used as a measure of boldness, a larger distance to the center indicating reduced boldness and vice versa (Burns 2008; Maximino et al. 2010). Hence, we compared the distance to the arena center between the telencephalon size selection lines. Distance to arena center (mm) was calculated as the median distance to center across all fish per shoal for every frame during the observation period, i.e. the 2nd to the 10th minute. This allowed us to control, at least to some extent, for potential differences in boldness during the observation period.

### Statistical analyses

We ran all analyses and generated all figures in the open-access software R (v 4.0.1, http://R-project.org/).

We examined how relative telencephalon size influenced collective motion in single-sex guppy shoals. Given the well-known sex differences in shoaling behavior in guppies (Croft et al. 2003b), we separated our analysis by sex. Hence, we fitted separate linear models (LM) to females and males and to the collective motion variables, by using the *lm* functions in the *stats* package. We modeled the response variable as a function of the explanatory variable telencephalon size_(small vs large telencephalon)_, with mean body size (centered at its mean) as a covariate in all models. Since alignment interacts with speed (Kent et al. 2019), we incorporated speed as a covariate in the model examining alignment score. We corrected for multiple testing by adjusting the significance levels via the Benjamini-Hochberg method (Haynes 2013). The artificial selection procedure was replicated 3 times (see Methods, section 2a). To account for any underlying differences between the 3 replicates, this variable should be fitted as a random effect nested in selection lines (Harrison et al. 2018). However, since random effects require at least 5 levels to accurately estimate among-group variance in the data (Harrison et al. 2018), we fitted replicate as a covariate and only retained it in the model if the effect was significant. After controlling for multiple testing, replicate was only retained in two models examining alignment and shoal spread in females.

We ran a separate linear model for the distance to the arena center for females and males. To test for differences in boldness between the telencephalon size selection lines, we fitted distance to the arena center as a function of the explanatory variable telencephalon size_(small vs large telencephalon)_ with mean body size (centered at its mean) and replicate (since the effect was significant) as covariates.

Response variables were either log, square root, or power transformed to meet the model assumptions when necessary. Please see the detailed R script on how all 12 separate response variables were transformed. The assumptions of normality and equality of variances for all linear models were confirmed by visual inspection of the residuals.

## Results

**Fig. 1.**
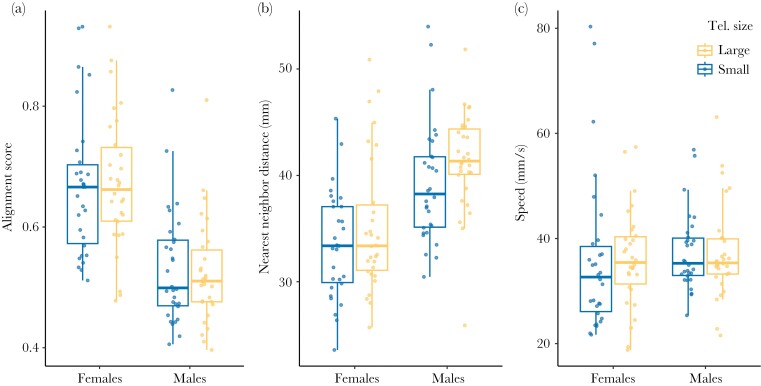
Boxplots of (a) alignment score, (b) distance to the nearest neighbor, and (c) speed for female and male guppy shoals of 8 individuals artificially selected for small (blue) and large (yellow) relative telencephalon size (Tel. size) assayed in an open-field test. Blue and yellow markers show the median value per guppy shoal during 8 min. Horizontal lines indicate medians, boxes indicate the interquartile range, and whiskers indicate all points within 1.5 times the interquartile range. *N* = 120.

**Fig. 2.**
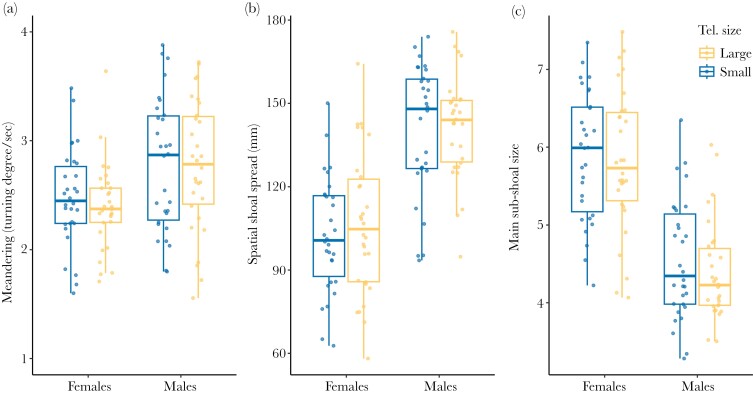
Boxplots of (a) meandering, (b) spatial shoal spread, and (c) main sub-shoal size for female and male guppy shoals of 8 individuals artificially selected for small (blue) and large (yellow) relative telencephalon size (Tel. size) assayed in an open-field test. Blue and yellow markers show the median value per guppy shoal during 8 min. Horizontal lines indicate medians, boxes indicate the interquartile range, and whiskers indicate all points within 1.5 times the interquartile range. *N* = 120.

We asked whether 6 aspects of collective motion, related to shoal structure and dynamics in guppies (Herbert-Read et al. 2017), differed between single-sex guppy shoals of 8 fish artificially selected for small and large relative telencephalon size. We examined alignment, attraction, speed, meandering, spatial shoal spread, and main sub-shoal size. We found that telencephalon size selection lines did not explain variation in these collective motion variables in female shoals, nor individuals in male shoals (Table 1, Figs. 1a–c and 2a–c). When swimming in an open arena, individuals from the large telencephalon size selection line were similarly aligned with shoal members as individuals from the small telencephalon size selection line in females and in males (Table 1). Across telencephalon size selection lines, attraction to the nearest neighbor was similar in females and in males (Table 1). Individuals from small and large telencephalon size selection lines explored the arena at similar swimming speed in females and in males (Table 1). The meandering rate (turning degree per second) was equal in individuals from the small and large telencephalon size shoals in females and in males (Table 1). Spatial shoal spread was similar in guppy shoals from the small and large telencephalon size selection lines in females and males (Table 1). On average, individuals from the large and small telencephalon size selection lines formed similar main sub-shoal sizes in females and males (Table 1).

**Table 1.** Reported are *t*-statistics and *P*-values (with *P*-values adjusted for multiple testing within brackets), as well as the regression slope estimates and their SE, from linear models evaluating the relationship between telencephalon (tel.) size selection lines and collective motion in guppies. Reported is the effect of the dependent variable telencephalon size. Reported are also the mean values and their SE. For full statistical output, see Supplementary Material.

	*t*	df	*P*-value	Estimate ± SE	Mean values ± SE	
					Large tel.	Small tel.
*Female shoals*						
Alignment score	0.35	54	0.73 (0.87)	0.004 (0.012)	0.67 ± 0.02	0.67 ± 0.02
Attraction (mm)	1.07	57	0.29 (0.87)	0.043 (0.040)	35.27 ± 1.17	33.48 ± 0.91
Speed (mm/sec)	0.46	57	0.65 (0.87)	0.037 (0.081)	36.17 ± 1.69	36.01 ± 2.73
Meandering	−0.60	57	0.55 (0.87)	−0.026 (0.044)	2.39 ± 0.07	2.48 ± 0.08
Shoal spread (mm)	0.47	55	0.64 (0.87)	0.026 (0.056)	106.59 ± 4.70	102.56 ± 3.79
Main sub-shoal size	−0.17	57	0.87 (0.87)	−0.219 (1.309)	5.83 ± 0.17	5.88 ± 0.15
*Male shoals*						
Alignment score	−0.53	56	0.60 (0.93)	−0.019 (0.037)	0.52 ± 0.02	0.53 ± 0.02
Attraction	1.93	57	0.06 (0.35)	0.062 (0.032)	41.35 ± 0.85	39.24 ± 1.02
Speed	−0.18	57	0.86 (0.93)	−0.010 (0.054)	37.26 ± 1.62	37.40 ± 1.33
Meandering	−0.09	57	0.93 (0.93)	−0.005 (0.059)	2.77 ± 0.11	2.78 ± 0.11
Shoal spread (mm)	0.35	57	0.73 (0.93)	0.083 (0.237)	141.55 ± 3.32	140.49 ± 4.30
Main sub-shoal size	−0.97	57	0.34 (0.93)	−0.037 (0.038)	4.40 ± 0.11	4.55 ± 0.14

Distance to arena center did not differ between the telencephalon selection size lines in females (LM: Estimate ± SE = 0.004 (0.01), *t *= 0.39, df = 55, *P *= 0.70), and in males (LM: Estimate ± SE = −0.003 (0.01), *t *= −0.28, df = 55, *P *= 0.78). This implies that there is no effect of telencephalon size selection lines in distance to arena center in females or in males.

For full details on statistical outcomes including covariates, see Supplementary Tables S1a–b and S2 and available R code.

## Discussion

This study represents the first experimental examination of the impact of variation in relative brain region size on different facets of collective movements. Our comprehensive analysis of 6 shoaling characteristics suggests that a 10% difference in relative telencephalon size does not contribute to variation in the measured aspects of collective motion assessed in the studied species.

None of the collective motion variables tested here were affected by the circa 10% differences in relative telencephalon size among these selection lines. These differences in telencephalon size correspond to telencephalon size differences found in natural populations (Fong et al. 2021) and should thus be biologically relevant. One possible explanation for our findings is that other brain regions play a dominant role in influencing collective motion. Kotrschal et al. (2020) artificially selected guppies on the level of alignment (Kotrschal et al. 2020). Microcomputed tomography on the polarization guppy selection lines revealed an enlargement of the thalamus and of the optic tectum but a reduction in the size of the medulla oblongata. No discernible differences were observed in 8 additional brain regions, including the telencephalon, compared to the control lines (Corral-López et al. 2023). This suggests that telencephalon size does not influence collective motion in female guppies, and our results support this finding. Furthermore, neural activity was recently found to increase in the preoptic area but not in 2 telencephalic sub-regions or in a sub-region of the ventral pallium, in female guppies exposed to large compared to small shoal sizes (Cabrera-Álvarez et al. 2017). Again, this indicates a minor importance of telencephalon in collective motion, at least in guppies. Although the telencephalon has been linked to collective motion in fish (Shinozuka and Watanabe 2004), and also other social behaviors in schooling and group living fish species (Lecchini et al. 2014; Fischer et al. 2015), we thus suggest that it is not a key area or that it has only a secondary role in causing variation in collective motion. We conclude that other brain regions may be more essential components of the neural circuity involved in the evolution of collective motion in fish. These could include the thalamus and optic tectum that regulates perception, attention, and motor responses, as recently proposed by Corral-Lôpez et al. (2023). Lesion studies have been highly important to reveal specific functions of distinct brain regions (e.g. Shinozuka and Watanabe 2004). However, lesions can also damage other relevant brain tissue, and they have limitations when it comes to revealing the functional aspects of evolutionary changes in behavior. Therefore, artificial selection can be an additional tool to investigate evolutionary functional aspects of brain morphology variation.

Collective movements are often explained by simple rules governing interactions among neighboring individuals (Couzin and Krause 2003; Couzin 2009; Sumpter 2010). Guppies subjected to artificial selection on relative brain size, with discernable differences in cognitive abilities (Kotrschal et al. 2013; Buechel et al. 2018), do not show differences in collective motion across brain size selection lines (Kotrschal et al. 2018). From this, Kotrschal et al. (2018) concluded that the simple interaction rules most likely stem from basic cognitive functions that do not require additional brain tissue for processing. The telencephalon, known as the cognitive center in fish (Portavella et al. 2002; Broglio et al. 2003, 2005; Salas et al. 2003), has been implicated in more advanced cognitive abilities also in the selection lines used in the current study (Triki et al. 2023). The absence of an effect of telencephalon size on collective motion in this study supports earlier findings suggesting that collective motion may not hinge on advanced cognitive abilities. Another aspect of these results is that they imply that the cognitive processes required to achieve collective motion may be decoupled from the cognitive processes involved in the social interactions required in complex group structures. Complex group structure is generally associated with increase in brain size or an increase in telencephalon size (Dunbar 1998; Reader and Laland 2002; Dunbar and Schultz 2007; Triki et al. 2020), and also with advanced cognitive abilities (Reader and Laland 2002; Amici et al. 2008; Ashton et al. 2018). Neither previous assays on selection lines with different brain size (Kotrschal et al. 2013, 2018), nor the present assays on selection lines with different telencephalon size have found any effects on collective motion. The lack of any effects of telencephalon size could be because we have not yet been able to generate enough divergence between the large and small telencephalon size selected lines. However, we have previously found that the existing divergence in telencephalon size affects several aspects of cognition (Triki et al. 2022, 2023). This includes several advanced cognitive abilities such as detour learning, reversal learning and working memory that were enhanced in the large compared to the small telencephalon size selection lines (Triki et al. 2022, 2023). We interpret these results such that divergence should be substantial enough to demonstrate differences in collective motion should they have existed. This further corroborates the hypothesis that collective motion is generated by basic cognitive functions, such as perception and attention (Corral-López et al. 2023). Whereas changes in telencephalon size change the processing of cognitive functions such as learning, memory, and decision-making (Triki et al. 2022, 2023).

Collective motion can be influenced by various environmental and social factors. For instance, the composition of the shoal, individual variation, external stimuli, or boldness can all contribute to the overall collective motion dynamics (Croft et al. 2003a, 2003b; Hertbert-Read 2016; Davis et al. 2017; Herbert-Read et al. 2017; Jolles et al. 2017). When swimming in an open arena, there was no difference in distance to arena center between the telencephalon size selection lines in females or in males. This suggests that differences in boldness between the artificial selection on telencephalon size selection lines are unlikely to hide any otherwise apparent differences in collection motion. It is possible that our use of single-sex shoals has affected the results. Guppies are living in a fission-fusion dynamic composed of both sexes in natural environments (Seghers 1974). At the same time, female guppies tend to form more stable groups, while males change between shoals more frequently (Croft et al. 2003b). Furthermore, no sex-specific effects of the artificial selection on telencephalon size have hitherto been found (Fong et al. 2021). We, therefore, think that it is unlikely that mixed-sex shoals would reveal differences in collective motion caused by telencephalon size.

## Conclusions

To conclude, variation in telencephalon size does not have any significant impact on variation in the shoaling dynamics that characterize collective motion in male or female guppy shoals. Future studies on the effects of telencephalon size on collective motion under more complex ecologically relevant conditions, for instance, during predation threat, could potentially further improve our understanding of the evolution of collective motion. Such analyses form the next step in the battery of assays planned for these selection lines.

## Supplementary Material

arae033_suppl_Supplementary_Materials

## Data Availability

Analyses reported in this article can be reproduced using the data provided by Boussard et al. (2024).
