# Supplementary material for: Relative telencephalon size does not affect collective motion in the guppy (Poecilia reticulata)
Source: Behav Ecol. 2024 May 3;35(4):arae033. doi: 10.1093/beheco/arae033 (PMC11110457; doi:10.1093/beheco/arae033)
Supplement: arae033_suppl_Supplementary_Materials [file arae033_suppl_supplementary_materials.docx]

**Supplementary material**

**Supporting Tables**

**Table S1.** Reported are t-statistics and p-values (with p-values adjusted for multiple testing within brackets), as well as the regression slope estimates and their SE, from linear models and linear mixed models evaluating the relationship between telencephalon size and collective motion in female guppies. Included are the dependent variable telencephalon size, as well as the covariates body size, speed when controlling for activity and replicate when the effect was significant. Df = 54 for a) Alignment, df = 57 for b) Attraction, c) Speed, d) meandering and f) sub-shoal size, and df = 55 for e) shoal spread. Small telencephalon size selection lines and replicate 1 are set as baseline. Significant p-values are highlighted in bold. N = 60.

1. Alignment

|  | t | p-values | Estimate ± SE |
| --- | --- | --- | --- |
| Telencephalon size | 0.35 | 0.73 (0.87) | 0.004 (0.012) |
| Body size | -1.39 | 0.17 (0.34) | <-0.001 (<0.001) |
| Speed | 10.25 | <0.001 | 0.005 (<0.001) |
| Replicate 1 | 0.45 | 0.66 (0.88) | 0.007 (0.015) |
| Replicate 2 | -2.80 | 0.007 (0.04) | -0.046 (0.016) |

1. Attraction

|  | t | p-values | Estimate ± SE |
| --- | --- | --- | --- |
| Telencephalon size | 1.07 | 0.29 (0.87) | 0.043 (0.040) |
| Body size | 2.33 | 0.02 (0.14) | 0.001 (<0.001) |

1. Speed

|  | t | p-values | Estimate ± SE |
| --- | --- | --- | --- |
| Telencephalon size | 0.46 | 0.65 (0.87) | 0.037 (0.081) |
| Body size | -0.24 | 0.81 (0.96) | <-0.001 (<0.001) |

1. Meandering

|  | t | p-values | Estimate ± SE |
| --- | --- | --- | --- |
| Telencephalon size | -0.60 | 0.55 (0.87) | -0.026 (0.043) |
| Body size | -2.00 | 0.05 (0.15) | <-0.001 (<0.001) |

1. Spatial shoal spread

|  | t | p-values | Estimate ± SE |
| --- | --- | --- | --- |
| Telencephalon size | 0.47 | 0.64 (0.87) | 0.026 (0.056) |
| Body size | 1.11 | 0.27 (0.55) | <0.001 (<0.001) |
| Replicate 2 | 1.40 | 0.17 (0.43) | 0.097 (0.069) |
| Replicate 3 | 3.00 | 0.004 (0.04) | 0.219 (0.073) |

1. Main sub-shoal size

|  | t | p-values | Estimate ± SE |
| --- | --- | --- | --- |
| Telencephalon size | -0.17 | 0.87 (0.87) | -0.219 (1.309) |
| Body size | -0.56 | 0.58 (0.87) | -0.008 (0.014) |

**Table S2.** Reported are t-statistics and p-values, as well as the regression slope estimates and their SE, from linear models and linear mixed models evaluating the relationship between telencephalon size and collective motion in male guppies. Included are the dependent variable telencephalon size, as well as the covariates body size, speed when controlling for activity. Df = 56 for a) Alignment, df = 57 for b) attraction, c) speed, d) meandering, e) shoal spread and f) sub-shoal size. Significant p-values are highlighted in bold. Small telencephalon size selection lines is set as baseline. N = 60.

1. Alignment

|  | t | p-value | Estimate ± SE |
| --- | --- | --- | --- |
| Telencephalon size | -0.53 | 0.60 (0.93) | -0.019 (0.037) |
| Body size | -0.95 | 0.35 (0.56) | <-0.001 (<0.002) |
| Speed | 4.24 | <0.001 | 0.009 (0.002) |

1. Attraction

|  | t | p-values | Estimate ± SE |
| --- | --- | --- | --- |
| Telencephalon size | 1.93 | 0.06 (0.35) | 0.062 (0.032) |
| Body size | 2.53 | 0.01 (0.08) | 0.002 (0.001) |

1. Speed

|  | t | p-values | Estimate ± SE |
| --- | --- | --- | --- |
| Telencephalon size | -0.18 | 0.86 (0.93) | -0.010 (0.054) |
| Body size | 0.66 | 0.51 (0.59) | <0.001 (0.001) |

1. Meandering

|  | t | p-values | Estimate ± SE |
| --- | --- | --- | --- |
| Telencephalon size | -0.09 | 0.93 (0.93) | -0.005 (0.059) |
| Body size | -0.54 | 0.59 (0.59) | <-0.001 (0.001) |

1. Spatial shoal spread

|  | t | p-values | Estimate ± SE |
| --- | --- | --- | --- |
| Telencephalon size | 0.35 | 0.73 (0.93) | 0.082 (0.237) |
| Body size | 0.90 | 0.37 (0.56) | 0.004 (0.005) |

1. Main sub-shoal size

|  | t | p-values | Estimate ± SE |
| --- | --- | --- | --- |
| Telencephalon size | -0.96 | 0.34 (0.93) | -0.037 (0.038) |
| Body size | -2.06 | 0.04 (0.13) | -0.002 (<0.001) |

**Table S3.** Reported are t-statistics from linear models evaluating the relationship between telencephalon size and distance to arena centre in female and male guppy shoals. Included are the dependent variable telencephalon size and the covariates body size and replicate. Degrees of freedom = 55. Significant p-values are highlighted in bold. Small telencephalon size selection lines, and replicate 1 are set as baseline. N_females_ = 60, N_males_ = 60.

| Female shoals | t | p-value | Estimate ± SE |
| --- | --- | --- | --- |
| Telencephalon size | 0.39 | 0.70 | 0.004 (0.01) |
| Body size | -2.12 | **0.038** | <-0.001 (<0.001) |
| Replicate 2 | 2.50 | **0.016** | 0.03 (0.013) |
| Replicate 3 | 2.15 | **0.036** | 0.028(0.013) |
| Male shoals |  |  |  |
| Telencephalon size | -0.28 | 0.78 | -0.003 (0.012) |
| Body size | -3.14 | **0.003** | <-0.001 (<0.001) |
| Replicate 2 | 3.24 | **0.002** | 0.049 (0.015) |
| Replicate 3 | 0.16 | 0.87 | 0.002 (0.015) |
